# Metabolomic Analysis of Liver Tissue from the VX2 Rabbit Model of Secondary Liver Tumors

**DOI:** 10.1155/2014/310372

**Published:** 2014-03-02

**Authors:** R. Ibarra, J-E. Dazard, Y. Sandlers, F. Rehman, R. Abbas, R. Kombu, G-F. Zhang, H. Brunengraber, J. Sanabria

**Affiliations:** ^1^Departments of Surgery, Case Western Reserve University, School of Medicine and University Hospitals, Case Medical Center, 11100 Euclid Avenue, Cleveland, OH 44106, USA; ^2^Departments of Nutrition, Case Western Reserve University, School of Medicine and University Hospitals, Case Medical Center, 11100 Euclid Avenue, Cleveland, OH 44106, USA; ^3^Center for Proteomics and Bioinformatics, Case Western Reserve University, School of Medicine and University Hospitals, Case Medical Center, Cleveland, OH 44106, USA; ^4^Department of Surgery, Cancer Treatment Centers of America, Chicago, IL 60099, USA

## Abstract

*Purpose.* The incidence of liver neoplasms is rising in USA. The purpose of this study was to determine metabolic profiles of liver tissue during early cancer development. *Methods.* We used the rabbit VX2 model of liver tumors (LT) and a control group consisting of sham animals implanted with Gelfoam into their livers (LG). After two weeks from implantation, liver tissue from lobes with and without tumor was obtained from experimental animals (LT+/LT−) as well as liver tissue from controls (LG+/LG−). Peaks obtained by Gas Chromatography-Mass Spectrometry were subjected to identification. 56 metabolites were identified and their profiles compared between groups using principal component analysis (PCA) and a mixed-effect two-way ANOVA model. *Results.* Animals recovered from surgery uneventfully. Analyses identified a metabolite profile that significantly differs in experimental conditions after controlling the False Discovery Rate (FDR). 16 metabolites concentrations differed significantly when comparing samples from (LT+/LT−) to samples from (LG+/LG−) livers. A significant difference was also shown in 20 metabolites when comparing samples from (LT+) liver lobes to samples from (LT−) liver lobes. *Conclusion.* Normal liver tissue harboring malignancy had a distinct metabolic signature. The role of metabolic profiles on liver biopsies for the detection of early liver cancer remains to be determined.

## 1. Introduction

The incidence of liver tumors is rising in the USA, and it represents the second most common malignancy of the GI tract [1]. Therapy relies on early tumor detection but up to 80% of patients are not eligible for surgery at the time of diagnosis due to advanced stage of disease or to a medical condition that prohibits surgery. Available tumor markers for liver cancer screening, that is, AFP, CA19.9 and CEA, lack high levels of sensitivity and specificity [2] making the early diagnosis of liver neoplasm a difficult challenge for the clinician. Serum metabolites related to oxidative stress are thought to be potential biomarkers for early detection of liver cancer [3–5].

Metabolomics is defined as the systematic quantitative measurement of time-related pluriparametric metabolic responses of multicellular systems to pathophysiological stimuli or genetic modification [4, 5]. It has been used to describe metabolic changes of all low-molecular-weight compounds present in biological samples of several malignant processes [6–12]. Metabolic profiling of urine samples by GC/MS techniques and on plasma by ^1^H nuclear magnetic resonance (NMR) from patients with primary liver tumors had shown changes mainly related to the glycolytic pathway and lipid metabolism [13–20]. Metabolite profile within the liver tumor significantly differ from the ones seen within the distant noninvolved liver tissue from the same individual [21, 22]. It was hypothesized metabolic profiling from liver tissue obtained by a biopsy may provide us with distinct signatures for the detection of early malignancy, at a stage not detectable by standard imaging modalities. The aim of the present study was to define the metabolic changes that occur in the liver during the early development of hepatic malignancies. For this purpose, we made use of the extensively studied VX2 rabbit model of secondary liver tumors developed by Shope and Hurst in 1933 [23–28]. We described a statistically significant distinct metabolic profile of liver tissue harboring early malignancy.

## 2. Materials and Methods

### 2.1. Animal Model

A syngenic graft tumor was obtained by injection of a VX2 cell line in the thigh muscle (*Vastus medialis*) of a New Zealand white male rabbit as described by Rous et al., 1952 [29]. The VX2 cell line was obtained from Dr. Exner laboratory (Case Western Reserve University School of Medicine, Cleveland, OH). Tumor was left to grow within the hosted muscle for 1 month; then it was harvested and minced into cubes of 1 mm^3^. Growths were stored at −80°C in Fetal Bovine Serum (Cambrex, East Rutherford, NJ) with 10% DMSO until implantation when tissue was thawed and washed 3 times in Hank's Buffered Salt Solution (HBSS). General chemicals and reagents were obtained from Sigma-Aldrich (St Louis, MO). All experiments were approved by the Institutional Animal Care and Use Committee (IACUC) of the Case Western Reserve University and were performed in accordance with their guidelines.

Adult New Zealand white male rabbits weighing 2.5–3 kg (Covance Princeton, NJ) were quarantined for 15 days in standard conditions with food (Rabbit Chow, NJ) and water *ad libitum* prior to any procedure. Preoperatively, rabbits were anesthetized with Xylazine (5 mg/kg), Ketamine (50 mg/kg), and Acetylpromazine (10 mg/kg) administered IM. Surgical site was shaved and swabbed with Betadine solution (Purdue Pharma, CT) and infiltrated subcutaneously (SC) with Marcaine 0.25% without epinephrine for local anesthesia. Antibiotic prophylaxis was given SC with Penicillin (50,000 units/kg) and Gentamicin (3 mg/kg) prior to the procedure and was continued once daily for 48 hours.

### 2.2. Experimental Design

Rabbits were first divided into two experimental groups, (i) the Treated Group (*n* = 5; 10 paired samples) underwent median laparotomy and surgical implantation of VX2 tumors in 2 separate sites (right medial and right posterior liver lobes). Each hepatotomy site was carefully closed and labeled with a 6 : 0 Prolene suture (Ethicon, NJ). The abdominal wall was closed in two layers. All experimental animals received equal tumor load (1 mm^3^ × 2 implants). (ii) The Control Group (*n* = 2; 4 paired samples) consisted of animals implanted with Gelfoam (Ethicon, NJ) using the same surgical methodology. Two weeks after surgery all rabbits underwent a second laparotomy. Biopsies of healthy liver tissue were taken separately from the right lobe either adjacent to the tumor implant (LT+) or adjacent to the Gelfoam implant (LG+) and from the left lobe either distant from the tumor implant (LT−) or distant from the Gelfoam implant (LG−). Therefore, in this experimental design, tissue samples were obtained from 4 experimental subgroups (animals with tumor implants = LT+/LT−, and animals with Gelfoam implants = LG+/LG−) as explained in Table 1.

For the purpose of these studies, the tumor Treatment Effect and biopsy Site Effect are the main effects referring to the treated versus control and to the adjacent versus distant comparisons, respectively. In short, this experimental design is a two-way factorial design, where main effects (treatment, site) and interaction effect (treatment × site) can be analyzed statistically, with repeated measures on *n* = 7 units (rabbits), amounting to a total of 2*n* = 14 paired observations as explained in Table 1.

### 2.3. Pathology Evaluation

Samples were immediately frozen on liquid nitrogen and labeled and stored at −80°C. The rest of the liver was surgically removed and perfused-fixed with 10% formaldehyde and 90% PBS at room temperature. All surgical procedures were performed under sterile conditions.

Postoperatively, 20 mL of normal saline (0.9% NS) was given for insensible water losses and Buprenorphine (0.1 mg/kg; Sigma, MO) was given SC twice daily for 48 hours for pain control. All animals were examined three times a day for 72 hours and twice a day afterwards until sacrificed 2 weeks after tumor implantation. In prior study tumor growth was recognized as early as 14 days after tumor grafting.

Liver tissue obtained from each experimental group was sliced and embedded in paraffin and stained with hematoxylin and eosin (H&E) for histological examination. Blinded slides were assessed by a pathologist for evidence of tumor development. Tumor size and volume were calculated from digital records using Digi3 Digital Binocular Microscope with DigiPro 3.0 software (LaboMed, CA).

### 2.4. Liver Tissue Preparation

Powdered frozen liver tissue (25 mg) was spiked with 5 nmol of heptadecanoic acid (C^17^) as internal standard and extracted with 2 mL of CH_3_CN/methanol (1 : 1 precooled at −12°C and degassed with nitrogen flow) using a Polytron homogenizer. The slurry was centrifuged at 3,800 rpm for 30 minutes at 4°C and the supernatant was separated and dried with nitrogen gas flow. The residue was oximated with 30 *μ*L of 15 mg/mL of methoxyamine hydrochloride in dry pyridine and incubated at 30°C for 90 minutes. Derivatization was finished with 70 *μ*L of N-methyl-N-trimethyl-silyl-trifluoroacetamide with 1% trimethyl-chlorosilane (MSTFA + 1% TMSC) and the mixture was incubated again at 37°C for 40 min. Samples were then submitted for spectrometric analyses.

### 2.5. Gas Chromatography-Mass Spectrometry (GC-MS)

GC-MS analyses were performed on an Agilent 6890 gas chromatograph interfaced to an Agilent 5973 mass spectrometer equipped with a Phenomenex ZB-5 MSi capillary column (30 m × 0.25 mm i.d., 0.25 *μ*m film thicknesses). Injection volume was 1 *μ*L in splitless mode. Injector temperature was set at 250°C and the transfer line at 275°C. The carrier gas was helium at a constant flow rate of 1 mL/min. The GC oven temperature was initially kept at 60°C for 1 min and increased at a rate of 10°C/min to a final temperature of 325°C held for 10 min. EI ion source temperature was set to 250°C and the MS quadrupole temperature to 150°C. Mass spectra were acquired in scan mode with a mass range of 45 to 800 *m/z*. Raw data were deconvoluted with the National Institute of Standards and Technology (NIST) Automated Mass Spectral Deconvolution and Identification Software (AMDIS). After spectral analysis and data processing of 113 signals, 56 signals could be identified in 80% of all samples. Identified signals were confirmed by our metabolomic library and the Fiehn library (Agilent Technologies Inc, Santa Clara, CA). For further quantification, the data was exported to the SpectConnect server (Massachusetts Institute of Technology, Cambridge, MA) [30]. The concentration of each metabolite was expressed as its relative peak area (divided by the area of the corresponding internal standard in the same chromatogram). All 56 identified metabolic compounds were further used for statistical analyses.

### 2.6. Statistical Analyses

All analyses were carried out using the R language and environment, a platform from the R project for statistical modeling, computing, and graphics (http://www.r-project.org/).

#### 2.6.1. Preprocessing of Features

Features (metabolites) were first log-transformed and then variance-stabilized and normalized by our recently developed “*joint adaptive mean-variance regularization*” procedure as previously described [31, 32]. This helps remove sources of systematic variation in the measured intensities (bias and variance due to experimental artifacts) and to ensure that the usual assumption of normality and homoscedasticity are met for statistical inference purposes.

#### 2.6.2. Principal Component Analysis

Principal component analysis (PCA) was carried out and results displayed as *scree plots* (determines the order and the number of principal components (PCs) accounting for the largest variance in the data) and as a 2D *biplot* (uses the first two PCs to display information about (i) the metabolites as indicated by their variance and covariances, and (ii) the relationship between samples as indicated by interindividual distances).

#### 2.6.3. Statistical Inference of Differential Metabolite Concentrations for Label-Free GC-MS Analyses

Statistical modeling was performed using a linear mixed-effect model of analysis of variance (mixed two-way ANOVA), fitted univariately to each individual variable (single metabolite concentration). For statistical inference, we used empirical Bayes methods and posterior estimators derived from them (moderated *F*-, *t*-, and *B* statistics) that have proven to result in greater statistical power [31–35] and to be useful for ranking variables in terms of evidence for differential expression [32, 35–38]. Information was borrowed by constraining the within-block correlations to be equal between variables and by using empirical Bayes methods to moderate the standard deviations between them [39]. These methods are particularly appropriate when only few samples are available, as is always the case in high throughput datasets [35].

#### 2.6.4. Reports for Label-Free GC-MS Analyses

Contrasts were built for each of the effects of interest, and coefficients were estimated accordingly. Variables were ranked in order of evidence of differential concentration. Corresponding *P*-values were adjusted for multiple testing using the positive FDR (denoted pFDR) [40], a recent extension of the False Discovery Rate (FDR) procedure of Benjamini-Hochberg [41] that is less conservative [40, 42]. Tables report top-to-bottom ranked metabolites (rows) from the model fit for each metabolite and contrast of interest. Each table consists of columns with the following information: the estimated log_2_-fold change or *M* log-ratio (*M =* log_2_ (FC)) for each individual metabolite in theeffect or contrast of interest. Moderated *t*- and *B*-statistics represent different measures of statistical significance. The moderated *t*-statistic corresponds to the usual *t*-statistic except that information has been borrowed across variables (metabolites), while the *B*-statistic is the empirical Bayes log_2_ of the posterior odds that the metabolite is differentially expressed. Finally raw and adjusted *P-*values are listed. Note that in every list all the metabolites are ranked by adjusted *P-*value and then by *B-*statistic.

#### 2.6.5. Power Analysis and Sample Size Calculation

**Figure 1:**
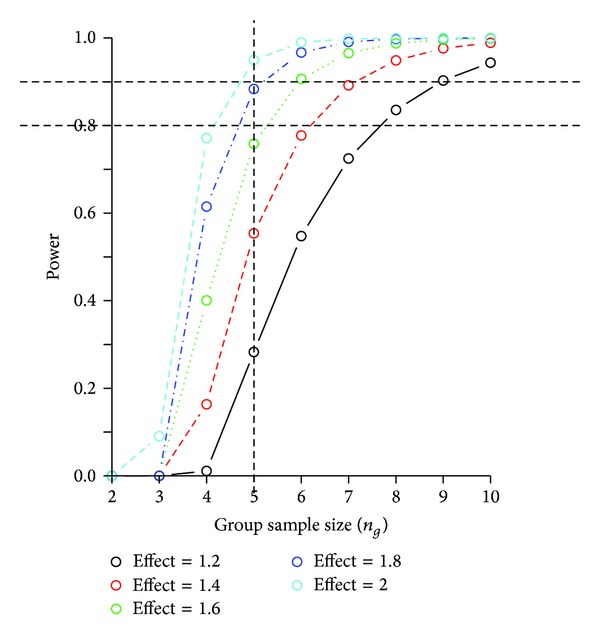
*Power analyses and sample size calculations*. The plots show statistical power curves (1 − *β*) for the main treatment effect (tumor versus control Gelfoam) as a function of group sample size (*n*_*g*_). Results are reported for a range of fixed fold change, for the fixed estimated median standard deviation common to all metabolites $\hat{\sigma }=1.00$, and for fixed ${\hat{\pi }}_{0}=0.92$, while controlling the False Discovery Rate at 10%. Results show that a group sample size of *n*_*g*_ = 5 in a balanced paired experimental design detects a twofold change in the effect of interest with more than 90% power.

We used the method described in Liu and Hwang [43] and the pFDR as described above. After variance stabilization and normalization of the data as described above [31, 32], the usual distributional assumptions of test statistics become applicable [43]. Under the above assumption and assuming a balanced paired design, denote the experimental group sample size by *n*_*g*_ (individual rabbits per group), the common standard deviation (to all metabolites) by *σ*, and the effect size by Δ/*σ*. We calculated the group sample size n_g_ required to detect, for example, a minimum fold change FC (Δ) in the treatment effect (tumor versus control Gelfoam) with at most *α*% FDR as a function of power 1 − *β*, the parameter *π*_0_ (interpreted as the probability of non-differentially expressed metabolites), the common standard deviation *σ*, and for a fixed level *α* of pFDR. Based on the data, parameters estimates were ${\hat{\pi }}_{0}\approx 0.88-0.96$ and $\hat{\sigma }\approx 0.95-1.05$, which is consistent with estimates found in other platforms in high dimensional data [43]. Under the above assumptions and estimations, for fixed ${\hat{\pi }}_{0}=0.92$ and $\hat{\sigma }=1.00$, while controlling the FDR at less than 10%, a group sample size as low as *n*_*g*_ = 5 can detect a twofold change on the transformed scale with more than 90% power (Figure 1).

## 3. Results

**Figure 2:**
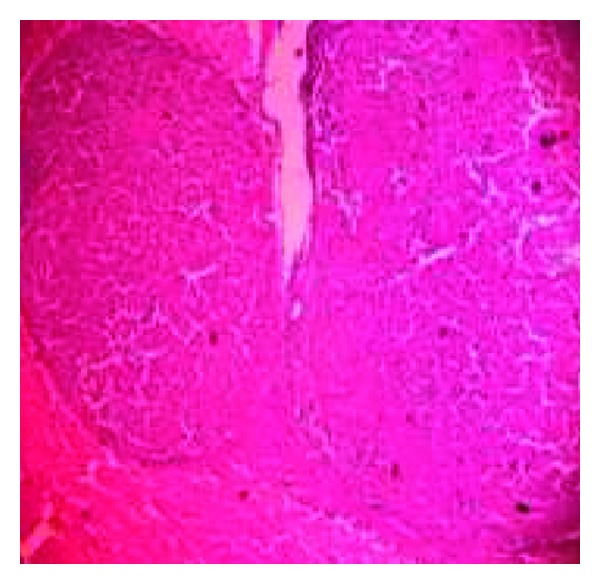
*Microscopic morphology of VX2 tumors*. All implanted tumor grafts showed macroscopic growth on liver sites. At histology, most tumors had a pseudocapsule formed by fibrotic tissue that surrounded the tumor which contained typical epithelial cells with malignant morphology without neoangiogenesis, tumor invasion, or lymphocyte infiltration at this stage of tumor growth (panel ×20).

Tumor grew in all experimental animals (Figure 2). All tumors were similar in size, with a maximum diameter of 8.4 ± 5.96 mm and a tumor volume of 241.8 ± 78.6 mm^3^. There was no evidence for tumor spread or distant metastasis by gross examination of the abdomen, chest, and brain. Animals from the control group presented normal livers without signs of the Gelfoam implant. All rabbits were clinically stable and no differences were noted regarding their food and water intake or their body weight.

**Figure 3:**
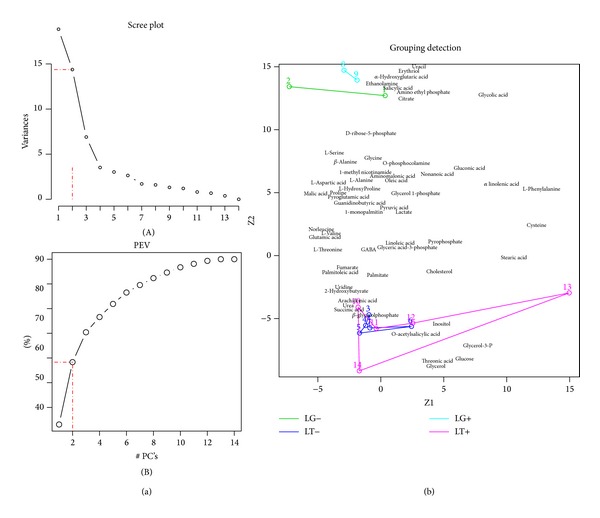
*Principal component analysis (PCA).* (a) PCA scree plots showing how much of variance is accounted for by each principal component (A) and that a minimum of two principal components ([Z1, Z2]) is enough to explain ~58% of the total variance (percentage of explained variance-PEV) in the data (B). (b) PCA biplot of samples and metabolites in the [Z1, Z2] principal component space. Note how the control group samples (LG+ and LG−) overlap with each other, as well as the treated group samples (LT+ and LT−) with each other. In addition, note the clear separation between the distant group samples (LT+ and LG+) and between the adjacent group samples (LT− and LG−).

Principal component analysis (PCA) showed that a minimum of two components explained at least 58% of the total variance of the data. Based on the metabolic concentration profiles, PCA was able to separate liver tissue samples of animals where tumor was implanted (LT+/LT−) from liver tissue samples of animals where Gelfoam was implanted (LG+/LG−) (Figure 3). Furthermore, PCA analysis shows that the liver tissue samples adjacent to the tumor could not be separated from the liver tissue samples distant from the tumor, whether this was observed in the treated samples (LT+/LT−) or in the Gelfoam control samples (LG+/LG−). This is indicative of an overall lack of site effect. In contrast, PCA analysis shows an overall strong treatment effect as evidenced by the clear separation of treated samples (LT+/LT−) from control samples (LG+/LG−). Also, this clear separation remained evident whether considering liver tissue samples adjacent to the tumor only (LT+ versus LG+) or liver tissue samples distant from the tumor (LT− versus LG−).

**Figure 4:**
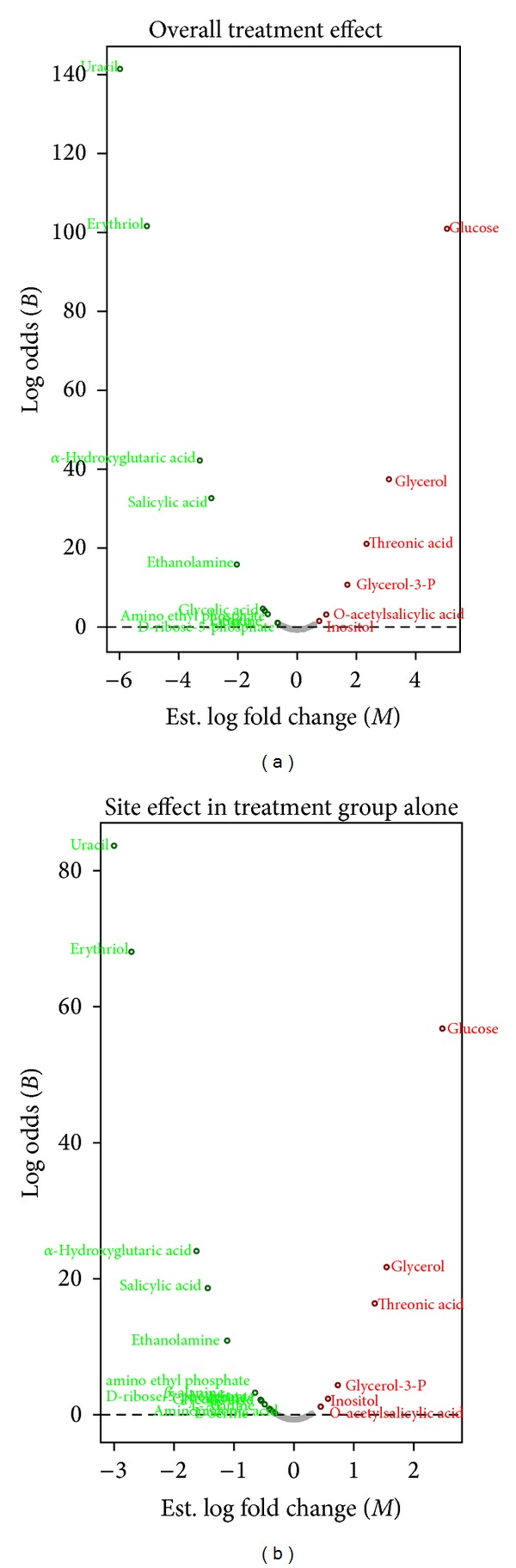
*t-test volcano plots*. (a) Overall treatment effect. (b) Site effect, conditioning on the Treated Group. The volcano plot is a scatter plot of all metabolite species arranged by an individual measure of magnitude of change of expression between experimental groups (horizontal axis) versus a corresponding measure of statistical significance (vertical axis). The horizontal axis represents the estimated log-fold-change of differential expression, denoted log_2_(FC) or *M*. The vertical axis represents the log-odds of differential expression, denoted log_2_(Odds) or *B*. The most significant metabolites are those that have the largest *M* in absolute value and the largest *B*. Metabolites (dots) whose relative concentrations are significantly downregulated (green) and upregulated (red) between experimental conditions are distributed in the upper left-hand side and upper right-hand side directions of the plot, respectively. The nonregulated metabolites are shown with grey dots. The horizontal dotted line represents a null log-odds of differential expression. FDR was controlled at 10%.

These results were further confirmed by ANOVA analyses: Table 2 lists the metabolic compounds whose concentrations were found statistically different in the treatment effect, that is, between the treated group (LT+/LT−) and the control group (LG+/LG−). A total of 16 identified compounds (10 downregulated, 6 upregulated) had False Discovery Rate-adjusted *P-*values (for multiplicity of testing) below the 10% FDR level. Interestingly, no statistically significant changes in metabolic compound concentrations were detected in the overall site effect, that is, between the experimental distant group (LT+ and LG+) and the adjacent group (LT− and LG−). To evaluate if this absence of significance could be due to a confounding effect of the Sham-treated samples over the tumor-treated samples, we analyzed the relative concentrations of metabolic compounds between adjacent versus distal (LT+ versus LT−) biopsy samples in the treated samples alone (Table 3). This comparison revealed a list of 20 identified compounds (14 down-regulated, 6 up-regulated) that had a FDR-adjusted *P-*values below the 10% FDR level. Graphical illustration of Tables 2 and 3 results are displayed in a space of statistical significance known as volcano plots (Figures 4(a) and 4(b), resp.).

## 4. Discussion

Recent studies focused on primary liver tumors that have aimed to describe metabolic fingerprints of tumor development. While plasma and urine samples [13, 15, 16] give the potential application of this technique for diagnosis and followup in a noninvasive approach, they are susceptible to variations of metabolites from possible simultaneous processes in the body and may not reflect the specific metabolic alterations of tumor growth. In contrast, analysis of tissue samples will only reflect the metabolic profile of the affected organ, providing us with a better understanding of the metabolic changes occurring at the tumor site. In this study we used a nontargeted GC-MS metabolomic approach to profile the changes present in the tumor periphery as well as the metabolic response of healthy nonadjacent liver tissue in the VX2 rabbit model of secondary liver tumors. Principal component analysis performed on metabolic profiles in liver tissue differentiated animals with tumor from Sham animals as early as 14 days after tumor grafting. We profiled 56 metabolites and most of our findings resemble previously described changes for HCC [17, 21, 22] and other tumors [6, 10, 44]. Changes are mostly related to the metabolism of carbohydrates, lipids, and amino acids and related to inflammation and oxidative stress. Relevant metabolites (Table 4) in liver metabolism and their profile under the influence of a growing tumor are discussed.

### 4.1. Carbohydrates

The high concentrations of glucose observed in samples from the experimental group when compared to Shams seems opposite of what has been described in the literature. In the tumor mass, an upregulation of glycolysis should use glucose as its principal substrate lowering its concentration. Yang et al. analyzed tumor tissue samples from 17 patients with HCC and compared them with the noninvolved adjacent liver tissue as a control [22]. They found a significantly lower concentration of glucose in the tumor samples, and this concentration was even lower in high grade tumors when compared to low grade tumors. Differences may be due to different sampling design; (i) in our study the tissue samples did not include the tumor mass and (ii) in the human study a group of healthy humans was not included as a control. In addition, the glucose uptake of cancer cells is about 30 times higher due to the increase expression of glucose transporters and hexokinase [45]. Our results showed an increase in the amount of glucose available for the hepatocytes in the healthy noninvolved liver that can be used by the highly active glycolytic metabolism induced by the tumor. The glycolytic disturbances found in this tumor model were accentuated in the healthy tissue close to the tumor when compared with the lobe without tumor, a finding probably due to the presence of a highly active tumor grown in need of energy substrates promoting a “stealing phenomenon” from the surrounding parenchyma and perhaps a relative state of starvation in distant but normal liver tissue.

Although the concentration of lactate was not significantly higher in samples of liver tissue adjacent to the tumor when compared to tissue samples distant from the tumor from the same animal group, a trend was noted. The classic finding in cancer, described by Warburg in 1930 [19], represents an increase in the rate of glycolysis with the final reduction of pyruvate into lactate by the enzyme lactate dehydrogenase (LDH) in order to regenerate the nicotinamide adenine dinucleotide (NAD+) necessary for the glycolytic pathway under an impaired oxidation of pyruvate in the mitochondria [18]. This effect is largely attributed to the activation of hypoxia-inducible factor-1b (HIF-1b) in tumor cells, which increases the expression of glucose transporters and glycolytic enzymes, resulting in an upregulation of glycolysis [4, 6, 7, 46]. A recent study of metabolites in plasma and urine from a rat model of diethylnitrosamine-induced HCC found a similar increase in lactate production in the presence of tumor and its level was related to HCC invasion and metastasis [45]. The acidic environment that surrounds the cancer cells has been recently related to a “reversed Warburg effect” in which lactate increases the metastatic potential of the tumor cell and 3-hydroxybutyrate (a ketone body) promotes tumor growth [47–49]. The lack of significance may be due to sample timing where the tumor has not reached enough size to influence surrounding liver tissue towards a more anaerobic metabolism or to a sample site where cells analyzed were not malignant and their anaerobic metabolism, which is enhanced in tumorigenic cells as mean of survival is not manifested in normal cells with established blood supply. Disturbances of the normal oxidative process is manifested as well by a significantly lower concentration of citrate and *α*-hydroxyglutaric acid from liver tissue adjacent to the tumor (LT+) when compared to liver tissue distant to the tumor (LT−); nevertheless, the present studies cannot elucidate if these changes are due to increase consumption, decrease production, or both.

### 4.2. Lipids

Many studies have noted disturbances of lipid metabolism in cancer cells [7, 14, 17]. Yang reported decreased lipid concentrations in tissue samples from HCC compared to noninvolved liver tissue, suggesting an increased utilization as an energy substrate via *β*-oxidation and as a substrate for the synthesis of cell membranes due to the demand of proliferating cells [22]. The decrease in the levels of ethanolamine, the second most common head group for membrane phospholipids, suggests increased utilization due to cell proliferation. Increased activity of the enzyme ethanolamine kinase has been reported in cancer cells and it has been attributed to the synthesis of phosphoethanolamine, a component of cell membranes [50]. Furthermore, we observed an increase in the levels of glycerol and glycerol-3-P in all samples from the experimental group when compared to the Shams suggesting an increase in triglycerides catabolism. Glycerol-3-P can fuel up glycolysis via dihydroxyacetone phosphate (DHAP). The above described changes suggested disturbances of lipid metabolism in normal hepatocytes induced by the presence of nearby immortal cells. Metabolic changes are mainly characterized by an increased *β*-oxidation and by the provision of substrates for cell membrane synthesis.

### 4.3. Amino Acids

Studies describe significant disturbances in the metabolism of several aminoacids, such as alanine, glycine, and glutamate [13, 21, 22]. In this study the concentration of L-serine, L-alanine, and glycine were significantly downregulated, while the concentration of glutamate was unchanged, which may be due to a balance between increase consumption for protein synthesis proposed by some and increase production through accelerated protein breakdown by others [8]. The levels of cysteine, a precursor of glutathione, were noted to have a trend to be decreased in the (LT+) tissue when compared to the noninvolved liver lobe (LT−). This difference may be explained by an increase synthesis of glutathione by the surrounding hepatocytes (LT+) to cope with oxidative stress. To support this finding, aminomalonic acid, a dicarboxylic acid derived from cysteine via *β*-elimination of the sulfur residue, was found to be significantly lower in (LT+) samples when compared to the (LT−) samples.

The levels of *β*-alanine (an amino acid that differs from *α*-alanine) were found to be decreased in samples from (LT+) tissue. *β*-alanine is a product of the degradation of dihydrouracil, which is an intermediate in the catabolism of uracil. The levels of uracil and the ribose ring D-ribose-5-P were also found to be decreased in the (LT+) samples. These findings suggest a status of accelerated cell proliferation, also found in plasma samples of patients with leukemia [44].

Two metabolites were noted to be regulated in liver tissue adjacent to tumor implantation (LT+). Although their concentration did not reach statistical difference when compared to liver tissue distant from tumor implantation (LT−), their relative different concentrations may have biological implications. 1-Methylnicotinamide (MNA) is a product of the catabolism of nicotinamide via enzymatic methylation by the enzyme nicotinamide N-methyltranferase (NNMT); this enzyme has been found to be upregulated in liver cirrhosis [51] and liver tumors [52]. In our study the levels of MNA were found to be higher in samples of tissue adjacent to the tumor (LT+) when compared to samples of tissue away from the tumor (LT−). It has also been shown that the increased catabolism of NAD^+^ will further stimulate the conversion of pyruvate to lactate to replenish the NAD^+^ pool required for glycolysis. Furthermore, MNA has also been linked with hypermethylation of DNA reducing the expression of tumor suppressor genes, thus promoting tumor cell growth [53] and prostacyclin dependent anti-inflammatory and antithrombotic effects [54, 55]. The regulation of liver GABA_A_ receptors plays an important role in the regulation of hepatocyte regeneration proliferation and in the pathogenesis of HCC [56], since their activation results in cell hyperpolarization inhibiting DNA synthesis. A transient hepatocyte depolarization has been reported after partial hepatectomy in rats [57]. An increase in DNA replication mediated by polyamines follows cell depolarization. Human HCC tissues have been found to be depolarized and have decreased GABA_A_-*β*3 receptor expression when compared to adjacent nontumor tissue [58]. Moreover, the restoration of membrane potentials resulted in a decreased proliferative activity and slower growth rates in human HCC cell lines [59]. In the present study we noted a trend towards a decreased concentration of GABA in (LT+) samples suggesting a depolarized state that facilitates cell proliferation.

Precursors of proinflammatory mediators have roles in many cellular processes, including inflammation, proliferation, and apoptosis. Their specific role in cancer is under investigation [60]. Threonic acid (not to be confounded with the amino acid threonine) is a metabolite of ascorbic acid and it further enhances the cellular uptake of ascorbic acid. This metabolite was found to be different between all groups (LT+, LT−, and Shams). Its role in liver metabolism in relation to cancer or oxidative stress remains to be determined.

The findings of our study must be interpreted in light of some limitations. The metabolic and redox status of an animal with a tumor growth may be a function not only of the tumor biology and its mass but also, among other factors, their nutritional status. Although the number of Sham animals was low, the metabolites in the right and left lobes were identical and did not differ from the metabolic profile of healthy rabbits already known (Fiehn library at Agilent Technologies Inc, Santa Clara, CA). Furthermore, the process of obtaining samples for metabolic profiling is an invasive one. In spite of these boundaries, the present study showed that metabolic disturbances in animals with early liver tumor growth can be detected and perhaps metabolic signatures of liver tumor growth may overcome the limitations of current biomarkers used in the clinical setting.

Metabolic changes found in the very early growth of a secondary liver tumor resemble the ones previously described in HCC [14–16], suggesting that these biochemical alterations are not specific to the biology of the tumor but to the response of the surrounding healthy liver tissue in response to a malignant growth with rapid cell proliferation in need of energy substrates. Changes observed in the healthy tissue adjacent to the tumor share metabolic characteristics typically found in neoplastic tissue; they may point to metabolic alterations that precede the morphological changes in the tumor surroundings suggested by others [15]. Metabolic signatures in tissue from subjects with tumor development could prove to be useful in the early detection of liver malignancies.

## 5. Conclusions

Principal component analyses of the metabolic profile from liver tissue differentiated animals with tumor when compared to animals without tumor as early as 14 days after tumor grafting. Different metabolic patterns were seen in tissue samples from healthy liver close to the tumor, healthy liver away from the tumor, and healthy liver from Sham animals. Further studies to correlate the metabolic profiles of tumor tissue and serum are required and could become a promising clinical tool for the early detection of liver tumors and their recurrence.

## Figures and Tables

**Table 1:** 

	Site effect	
	Distant (−)	Adjacent (+)
Treatment effect		
Tumor-implant *Treated Group * (LT)	(LT−)	(LT+)
	Rabbit #1	Rabbit #1
	Rabbit #2	Rabbit #2
	*⋮*	*⋮*
	rabbit #5	rabbit #5

Gelfoam-implant *Control Group * (LG)	(LG−)	(LG+)
	Rabbit #6	Rabbit #6
	Rabbit #7	Rabbit #7

**Table:** (a) 10 downregulated

Downregulated				
	ID	*M* = log⁡_2_⁡(FC)	FDR Adj.	*B* = log⁡_2_⁡(Odds)
			*P* values	
1	Uracil	−5.9822	4.52*E* − 53	141.4358
2	Erythriol	−5.0750	9.78*E* − 41	101.6021
3	*α*-Hydroxyglutaric acid	−3.2867	2.28*E* − 19	42.2173
4	Salicylic acid	−2.8973	1.18*E* − 15	32.6557
5	Ethanolamine	−2.0405	1.38*E* − 08	15.8544
6	Glycolic acid	−1.1588	1.58*E* − 03	4.6512
7	Amino ethyl phosphate	−1.0952	2.72*E* − 03	4.0824
8	Citrate	−0.9941	6.46*E* − 03	3.2435
9	L-Serine	−0.6642	6.86*E* − 02	1.0709
10	D-ribose-5-phosphate	−0.6495	7.08*E* − 02	0.9943

**Table:** (b) 6 up-regulated

Up-regulated				
	ID	*M* = log⁡_2_⁡(FC)	FDR Adj.	*B* = log⁡_2_⁡(Odds)
			*P*-values	
1	Glucose	5.0586	1.08*E* − 40	100.9430
2	Glycerol	3.0981	1.55*E* − 17	37.4367
3	Threonic acid	2.3402	8.15*E* − 11	21.0678
4	Glycerol-3-P	1.6936	2.54*E* − 06	10.7094
5	O-acetylsalicylic acid	0.9825	6.62*E* − 03	3.1519
6	Inositol	0.7428	4.27*E* − 02	1.5101

**Table:** (a) 14 downregulated

Down-regulated				
	ID	*M* = log⁡_2_⁡(FC)	FDR Adj.	*B* = log⁡_2_⁡(Odds)
			*P* values	
1	Uracil	−3.00112	3.83*E* − 35	83.65428
2	Erythriol	−2.70968	9.69*E* − 30	68.06919
3	*α*-Hydroxyglutaric acid	−1.626	2.75*E* − 12	24.07396
4	Salicylic acid	−1.43637	4.63*E* − 10	18.63641
5	Ethanolamine	−1.11231	1.17*E* − 06	10.90255
6	Amino ethyl phosphate	−0.64698	0.004687	3.236894
7	D-Ribose-5-phosphate	−0.556	0.013184	2.212044
8	Citrate	−0.54086	0.014689	2.056548
9	*β*-Alanine	−0.48647	0.025933	1.533321
10	Glycolic acid	−0.41113	0.050703	0.900165
11	Glycine	−0.39026	0.058594	0.743548
12	L-Alanine	−0.33772	0.085378	0.385349
13	L-Serine	−0.33726	0.085378	0.382457
14	Aminomalonic acid	−0.31862	0.095196	0.267971

**Table:** (b) 6 up-regulated

Up-regulated				
	ID	*M* = log⁡_2_⁡(FC)	FDR Adj.	*B* = log⁡_2_⁡(Odds)
			*P*-values	
1	Glucose	2.477314	1.30*E* − 25	56.78349
2	Glycerol	1.546767	2.37*E* − 11	21.72003
3	Threonic acid	1.349669	4.13*E* − 09	16.37445
4	Glycerol-3-P	0.733706	0.001424	4.35813
5	Inositol	0.56952	0.012116	2.354559
6	O-acetylsalicylic acid	0.447728	0.037053	1.194456

**Table 4:** Relevant metabolites and their relative variation between liver tissue samples from adjacent (LT+) and distant (LT−) to tumor implantation site.

Metabolites	Adjacent to tumor (LT+)	Distant to tumor (LT−)	Type related mechanism
Glucose	↑	—	Increase glucose uptake
Lactate	↑	—	Increased *β*-oxidation—increased glycolysis
Glycerol	↑	—	Lipid metabolism
Glycerol-3-phosphate	↑	—	Increased lipids catabolism
Threonic acid	↑	—	Vitamin C metabolite
Inositol	↑	—	Oxidative stress—glutathione synthesis
Ethanolamine	↓	—	Cell replication—membranes
L-serine/L-alanine/glycine	↓	—	Protein synthesis
*β*-alanine	↓	—	Cell replication—uracil catabolism
Aminomalonic acid	↓	—	Oxidative stress—cysteine metabolism
Citrate	↓	—	Citrate cycle—oxidative-redox mitochondrial status
D-Ribose-5-phosphate	↓	—	Purine synthesis
Uracil	↓	—	Cell replication
1-methyl nicotinamide	↑	—	Nicotinamide catabolism—NNMT activation
GABA	↓	—	Cell replication

↑: for relative increase; ↓: for relative decrease; —: for no relative change. LT+: healthy liver tissue adjacent to the tumor and LT−:healthy liver tissue distant from the tumor.

## Abbreviations

HCC: Hepatocellular carcinoma
AFP: *α*-Fetoprotein
CA19.9: Carbohydrate antigen 19.9
CEA: Carcinoembryonic antigen
GS-MS: Gas Chromatography-Mass Spectrometry
IM: Intramuscular
DMSO: Dimethylsulfoxide
HBSS: Hank's Buffered Salt Solution
PBS: Phosphate buffered saline
SC: Subcutaneous
NS: Normal saline
H&E: Hematoxylin and eosin
MSTFA: N-Methyl-N-trimethyl-silyl-trifluoroacetamide
TMSC: Trimethyl-chlorosilane
NIST: National Institute of Standards and Technology
AMDIS: Automated Mass Spectral Deconvolution and Identification Software
LDH: Lactate dehydrogenase
NAD^+^: Nicotinamide adenine dinucleotide
HIF-1b: Hypoxia-inducible factor-1b
MNA: 1-Methylnicotinamide
NNMT: Nicotinamide N-methyltranferase
G3P: Glycerol-3-phosphate
DHAP: Dihydroxyacetone phosphate
IRP2: Iron regulatory protein 2
GABA: *γ*-Aminobutyric acid.
